# Advances in Laser Therapy for Hidradenitis Suppurativa: A Systematic Assessment of Current Evidence

**DOI:** 10.3390/jcm14217683

**Published:** 2025-10-29

**Authors:** Michał Gawroński, Kinga Bukowczyk, Julia Chęcińska, Julita Krupiczowicz, Michalina Kołomyjec, Magdalena Łyko, Alina Jankowska-Konsur

**Affiliations:** 1 Student Research Group of Experimental Dermatology, University Centre of General Dermatology and Oncodermatology, Wroclaw Medical University, Borowska 213, 50-556 Wrocław, Poland; michal.gawronski@student.umw.edu.pl (M.G.); kinga.bukowczyk@student.umw.edu.pl (K.B.); julia.checinska@student.umw.edu.pl (J.C.); julita.krupiczowicz@student.umw.edu.pl (J.K.); michalina.kolomyjec@student.umw.edu.pl (M.K.); 2 University Centre of General Dermatology and Oncodermatology, Wroclaw Medical University, Borowska 213, 50-556 Wrocław, Poland; alina.jankowska-konsur@umw.edu.pl

**Keywords:** hidradenitis suppurativa, acne inversa, treatment, laser, laser therapy

## Abstract

**Background**: Hidradenitis suppurativa (HS) is a chronic, recurrent skin disease that significantly impairs patients’ quality of life both physically and mentally. It often requires a complex treatment process. Laser therapy, which is highly effective and well-tolerated, is an effective alternative to pharmacological treatment. This review aimed to synthesize information on laser therapy for HS, highlighting its clinical outcomes. In the current management guidelines for hidradenitis suppurativa, laser therapy is listed as one of the recommended procedural treatment options, applicable at different stages of disease severity (Hurley I–III). **Methodology**: This systematic review was conducted using the PubMed and Embase databases, regardless of publication year, in accordance with the PRISMA guidelines. Applied key search terms were: “laser AND (hidradenitis suppurativa OR acne inversa)”. A total of 26 relevant studies were identified, and their data were extracted. **Results**: The CO_2_ laser is mainly used in advanced stages of the disease (Hurley II–III). It allows effective removal of lesions with a minimal risk of relapse and a good aesthetic effect. The Nd:YAG (neodymium-doped yttrium aluminum garnet; Nd: Y_3_Al_5_O_12_) laser is effective at various stages of the disease (Hurley I–III) by reducing inflammation and destroying hair follicles, thereby reducing disease symptoms. IPL (intense-pulse light) therapy, or the combination of IPL with radiofrequency (RF), known as LAight^®^, delivers significant clinical improvement and enhanced quality of life, especially in less advanced cases. The diode laser works precisely and deeply, leading to the selective destruction of hair follicles and fistulas. The Alexandrite laser (755 nm) also limits hair follicle occlusion and is particularly effective in patients with lighter skin phototypes. **Conclusions**: In modern dermatology, laser therapy is a reliable treatment for HS, contributing to effective regression of the disease at all stages. Combination strategies seem to improve clinical outcomes and enable a more personalized approach to HS, which is essential as various factors influence therapeutic efficacy. Further, larger-scale studies are needed to validate long-term outcomes and establish clinical guidelines.

## 1. Introduction

Hidradenitis suppurativa (HS) is a chronic, recurrent, autoinflammatory skin disease classified among neutrophilic dermatoses. It is characterized by the formation of extensive abscesses, fistulas, and scarring. Lesions most commonly occur in areas of the body rich in apocrine glands, including the anogenital region, groin, armpits, and under the breasts. The prevalence of HS in the general population is estimated at 0.99%. Statistically, women are more frequently affected than men [1].

The disease significantly reduces patients’ quality of life due to the associated pain caused by the formation of abscesses and scarring, which limits flexibility in flexural areas and also negatively affects mental health [2].

Treatment may be either pharmacological or surgical. Despite significant advances in the pharmacological treatment of HS with antibiotics, retinoids, hormonal therapies, and biologics, management may still prove insufficient, with only partial or temporary effectiveness. In clinical practice, patients often decide to discontinue therapy due to a lack of satisfactory results or unpleasant side effects, especially with long-term use of antibiotics or biologics, thus prompting the need for surgical methods. These include laser therapy, which has shown significant clinical improvement, particularly in advanced stages of the disease (Hurley stages II and III) [3]. For laser therapy to be effective, it is essential to determine the stage of the disease in order to select the most appropriate and effective laser treatment. Ultrasound imaging allows for precise diagnostics, as well as detailed assessment of the morphology and extent of skin lesions [4]. In the current management guidelines for hidradenitis suppurativa, laser therapy is listed as one of the recommended procedural treatment options, applicable at different stages of disease severity [3]. In this systematic review, we aimed to compile the various types of lasers used in the treatment of HS and summarize their therapeutic effects.

## 2. Materials and Methods

### 2.1. Search Strategy

**Figure 1:**
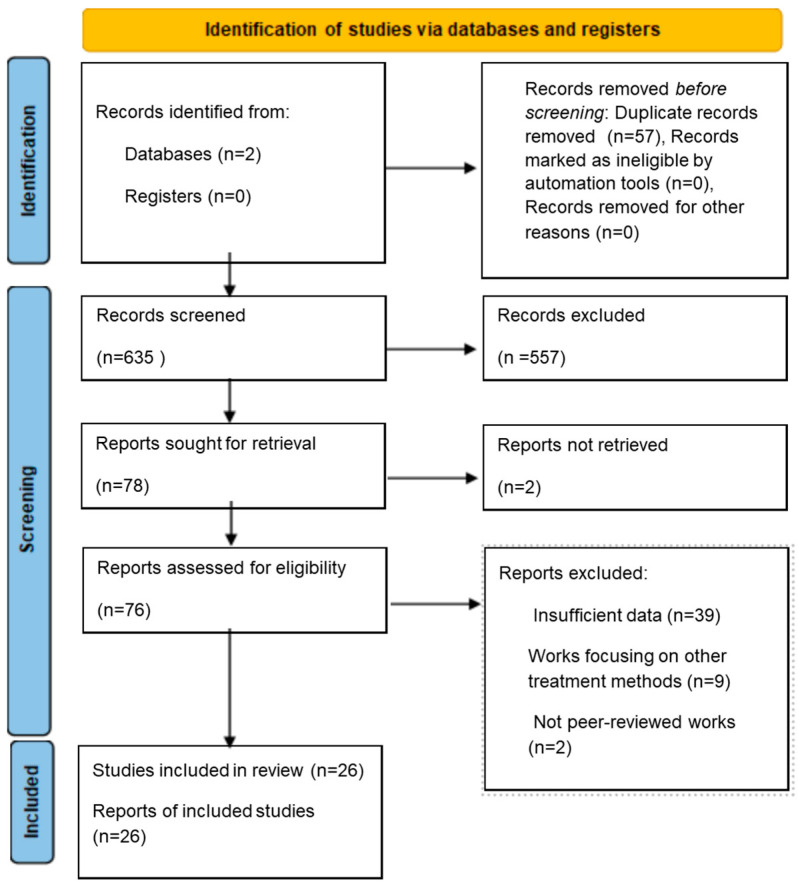
Search strategy.

A comprehensive literature search was conducted using the PubMed and Embase databases, which together cover the vast majority of biomedical and dermatology-related journals. However, additional databases such as Scopus or Web of Science were not included, as these databases mainly overlap with PubMed and Embase in terms of indexed dermatological research. The review protocol was not pre-registered; however, the methodology strictly followed the PRISMA 2020 guidelines to ensure transparency and reproducibility (Tables S1 and S2). All records, irrespective of publication year, were included in the search. The following search term was applied: “laser AND (hidradenitis suppurativa OR acne inversa).” Only studies published in English were included during the selection process. The last search was completed in April 2025. The whole search strategy and selection process are detailed in the PRISMA flow diagram (Figure 1).

### 2.2. Selection Criteria

Studies were included in the review if they met all of the following hierarchical criteria:

1. Written in English.
2. Original scientific research published in peer-reviewed journals.
3. Clearly defined study objectives, methodology, and results.

Only studies evaluating the use of laser therapy in the treatment of hidradenitis suppurativa were included. The selection process was conducted independently by two reviewers: M.G. and J.C. Before selection, both reviewers were familiarized with the inclusion criteria to ensure consistency. Any discrepancies in study eligibility were resolved through discussion within the research team.

### 2.3. Data Extraction

Data extraction was performed using a standardized data collection form in accordance with the PRISMA 2020 checklist. This systematic review was not registered in PROSPERO or any other database. Preliminary extraction was carried out by J.K. and independently verified by M.G. to ensure accuracy and consistency.

For each included study, the following information was extracted: the first author’s surname, year of publication, and type of study. Furthermore, detailed data were collected on the study objective, treatment protocol, description of the study sample, population characteristics, primary outcomes, type of laser used, and peer-review status. Any discrepancies identified during the data extraction phase were resolved through consensus among the research team.

A total of 692 papers were subjected to an initial screening conducted by J.K and M.K. As a result, 78 papers were selected for a second screening performed by M.G. and J.C. Next, reanalysis of the studies according to exclusion criteria was performed by K.B and M.G. Inter-rater reliability was assessed using Cohen’s Kappa coefficient, which showed substantial agreement between reviewers (κ = 0.79). Ultimately, 26 articles were included for further analysis.

### 2.4. Outcomes

Outcomes were analyzed qualitatively due to insufficient quantitative data. The quantitative data were reproduced, as only a few studies provide results, and their methodologies need to be more consistent to meta-analyze them.

## 3. CO_2_ Laser

CO_2_ laser therapy has become one of the promising surgical methods for the treatment of HS, especially in patients with severe, chronic, and recurrent forms of the disease. Several clinical studies have shown the efficacy of this technique, which consists of vaporization of lesions until healthy residual subcutaneous fat or fascia is reached, and healing by both primary and secondary intention. A comparison of the studies discussed below on the HS treatment using CO_2_ laser is provided in Table 1.

In one of the pioneering studies, Lapins et al. [5] evaluated the efficacy of the CO_2_ laser using the horizontal vaporization method to remove all affected tissue. Twenty-four patients diagnosed with HS, Hurley stage II, were chosen. The patients had a chronic course of the disease and recurrent abscesses, defined as more than three episodes per year. The selected anatomical regions were treated once, and wounds were allowed to heal by secondary intention. During a mean follow-up of 27 months, recurrence in the treated area occurred in 2 patients. In 4 patients, new lesions appeared 5–10 cm from the operation scars, in 10 patients, recurrences appeared in different anatomical regions. Eight patients remained recurrence-free. The healing time was on average 4 weeks, and the method was characterized by high patient acceptance and good cosmetic effect [5].

Similar results were obtained by Finley and Ratz [6], who performed 12 procedures of CO_2_ laser ablation in 7 female patients with HS in the axillary and inguinal-perineal regions. The wound surface area ranged between 4 and 60 cm^2^, and the average time for the wounds to heal was 6 weeks. During the follow-up period of 18.5 months, recurrence in the postoperative scar was noted in one case, and new lesions appeared in the untreated area in five patients. The patients were satisfied with the therapeutic effect. Complications included temporary paresthesias and axillary stricture in one patient and inguinal candidiasis in another. The authors emphasized that the use of a CO_2_ laser facilitates the precise removal of the affected tissue while ensuring hemostasis and sterilization of the surgical site [6].

In the study by Lapins et al. [7], an improved scanner-assisted CO_2_ laser ablation technique was used, which allows for more precise and controlled removal of the affected tissue in the axillary and inguinal-gluteal-perineal area. Each affected area was treated once. The 34 patients with Hurley stage II had HS for a mean time of 13.4 years and more than three recurrent episodes of abscesses per year before inclusion in the study. The mean healing time was 4 weeks, and during the follow-up period of 34.5 months, recurrence at the treatment site occurred in 4 patients, and eight had no recurrences. Twenty-five patients had flares in different anatomical regions, and in 12 patients, new lesions developed outside the surgical site, which the authors interpreted as a feature of natural disease progression rather than a failure of the procedure. An injury to an axillary nerve was suspected in one patient as they complained of 6-month-lasting paraesthesia after procedure. Most patients assessed the treatment results as significantly better than before the procedure, and the scars were aesthetically acceptable [7].

Further studies have confirmed the efficacy of CO_2_ laser therapy in patients with more advanced and refractory HS. Madan et al. [8] evaluated the use of CO_2_ laser in 9 patients with severe, multifocal HS who had failed to respond to previous therapies, an average of 4.5 different methods, both pharmacological and surgical. In the study, 27 laser treatments on the axilla and groin region were performed in 19 sessions. Lesions made of abscesses, sinuses, and granulation tissue were completely excised; where possible, the wounds were closed by primary intention or left to heal by secondary intention. After a mean healing time of 2 weeks, during a 12-month follow-up period, 6 of 9 patients achieved complete remission of lesions. Two patients complained of active HS at untreated sites in the same region as the scars, and one patient experienced discharging lesions in the postoperative scar after 3 months, which were successfully treated with another laser procedure. The main complication was a contracture scar in the axilla in two patients, which, however, did not limit limb mobility. High patient satisfaction was confirmed by a survey, with an average score of 8.5/10, and eight of nine patients would recommend this method to other patients [8].

Hazen and Hazen [9] evaluated CO_2_ laser excision with marsupialization in 61 patients with chronic, recurrent disease. The patients had advanced lesions with the presence of sinus tracts, scars, and chronic abscesses, and previous pharmacological and surgical therapies had been ineffective. A total of 154 procedures were performed in the following areas: groin, buttock, perirectal, axilla, inframammary or chest, abdomen, legs, neck, pilonidal sinus, and scalp. Multiple procedures were necessary, especially in obese and African American patients (average 3.8 treatments per patient), who had more extensive lesions and were more challenging to treat. The procedure consisted of excision of the affected tissues with a CO_2_ laser. The procedure was followed by vaporization of the base and edges of the surgical field, which created a smooth, pocket-like defect or plane. The wounds were allowed to heal by secondary intention, except for one patient who required suture closure. 2 weeks after surgery, they experienced dehiscence of the wound twice. The mean healing time was 8.8 weeks, and all patients achieved aesthetically and functionally acceptable results. Relapse of the disease in the treated areas was noted in only two patients. The most common complication was excessive postoperative granulation tissue, which responded well to treatment with topical silver nitrate. Three patients experienced postoperative cellulitis, and one patient developed Sweet’s syndrome, which resolved after corticosteroids. Follow-up showed an average of 4.1 years recurrence-free in treated areas. The authors emphasized that CO_2_ laser excision and marsupialization enable effective treatment of advanced HS lesions in an outpatient setting, with good patient tolerance and a high level of safety [9].

Crocco et al. [13] described the use of CO_2_ laser surgery in three patients with Hurley stage III. All patients had multifocal lesions with the presence of fistulas and scars, and no improvement after previous pharmacological and surgical therapies. The procedures were performed in an outpatient setting, using the CO_2_ laser. After the procedure, the wounds were left to heal by secondary intention. In one case, the procedure was repeated three times with 10-week intervals. In all three cases, complete healing of the lesions was achieved, in one patient within 9 weeks of the procedure, and the rest were unspecified. In one patient with axillary scar contracture, limb mobility improved after treatment. The authors concluded that the possibility of performing it in an outpatient setting, without the need for hospitalization, is a significant advantage of this technique [13].

In a comparative, multicenter, retrospective study, Sechi et al. [17] reported that the average healing time in HS Hurley stage II is significantly shorter after laser therapy (4.7 weeks) than after surgical deroofing (10.9 weeks). Furthermore, patients reported less severe pain, which may enhance their overall comfort and satisfaction after therapy [17].

A separate aspect of HS treatment is the management of post-inflammatory scars, which can persist despite the control of active disease. It may significantly worsen the patients’ quality of life. An innovative approach to scar treatment was proposed by Krakowski et al. [11], who reported the use of a fractionated 10,600 nm laser for treating cribriform scars in a teenage patient after HS in the intermammary region. This method involves selective photothermolysis that entails the creation of microscopic deep columns of thermal damage over a fraction of the treated area. Intact skin between the columns mediates a wound-healing and remodeling response, which is stimulated by mediators such as heat shock proteins, growth factors, metalloproteinases, and microRNAs. The study suggests that CO_2_ laser resurfacing may be a promising and safe option for treating post-HS scarring. Although these are preliminary observations based on a single case, they indicate the potential of this technology in improving aesthetic aspects. However, further controlled studies are needed to assess the safety and effectiveness of this method in the broader patient population [11].

In patients with HS, one of the complications may be obesity. Because patients with HS are affected by abscesses, fistulas, and scarring in the axillary and groin regions, their mobility in these areas is reduced, which may limit physical activity. Nicholson et al. [14] emphasize the significance of scar treatment to improve the patient’s range of motion and accelerate healing of chronic ulcerations. They presented a case of a 21-year-old obese female with stage III HS. After several failed treatment lines with oral and topical medications, including an incomplete response to Nd:YAG laser therapy, the patient underwent CO_2_ laser excision to the left axilla. During recovery, chronic ulceration worsened with movement, and scar contracture occurred. After 9 months of two non-healing ulcers, the patient underwent fractional ablative CO_2_ laser treatment in the surrounding areas. The therapy consisted of 4 sessions every 5–6 weeks with a fractional laser. After the final treatment, the ulcers disappeared, the scar structure was revised, and the significant decrease in tension around the ulcer edges accelerated healing. Importantly, since CO_2_ excision the patient lost 14% of her body weight as a result of mobility improvement [14].

Mikkelsen et al. [12] indicated the factors affecting the recurrence frequency of lesions in HS after CO_2_ laser therapy. They conducted a retrospective study involving 58 patients. Almost 30% of patients reported a lesion relapse in the same area, on average, after 12 months. However, 90% of patients would recommend this therapy to others (28% of them had a recurrence). Authors emphasized that obesity correlates with disease frequency and severity, thereby negatively affecting the effectiveness of CO_2_ laser treatment. Therefore, overweight HS patients should be strongly encouraged to lose weight as an adjunct to therapy [12].

A randomized, single–blinded right-left intraindividual controlled study by Abdel Azim et al. [15] evaluated the combined use of fractionated CO_2_ laser and long-pulse Nd:YAG laser in patients with Hurley stage I (10 patients) and II (10 patients). The study randomized 20 adult patients (55% women; mean age, 29.7 years) with bilateral, almost symmetric lesions who completed the analysis. The patients’ duration of HS ranged from 2 to 10 years. Four treatment sessions were performed at 2-week intervals. One side of the patient’s body (control side) was treated with Nd:YAG laser only, while the other side (test side) received a combination of fractionated CO_2_ laser and then Nd:YAG laser in the same session. Ice packs were instantly applied after the treatment. The results showed a clear advantage of the combined treatment. The mean degree of improvement, assessed by the physician global assessment (PGA) scale, in the side treated with the combination, was 90%, with a mean patient satisfaction score (VAS) of 8.05/10. For the side treated with Nd:YAG alone, the mean degree of improvement was 70.7%, and VAS 7/10. 2 weeks after the last treatment, complete resolution of lesions was achieved in 80% of patients with combined treatment, and the durability of improvement at 3 months was maintained in 55% (no relapse). For the side treated with Nd:YAG alone, the corresponding values were 35% and 20%, respectively. Histopathological examinations showed a greater reduction in inflammatory infiltration and skin edema after combined treatment compared to Nd:YAG alone. Importantly, no serious adverse events were reported besides the transient erythema that disappeared within 48 h. The authors suggest that a fractionated CO_2_ laser could increase Nd:YAG laser penetration by creating microscopic vertical channels lined with coagulated tissue and thereby reducing light scattering. Additionally, CO_2_ laser showed anti-inflammatory and collagen-remodeling effects, which could improve skin structure and reduce recurrences. Factors negatively affecting treatment efficacy included obesity, previous surgical procedures, and high PGA scoring. However, the use of combined treatment reduced the impact of these factors. The study provided clinical evidence of the advantage of combined therapy over Nd:YAG laser alone, while indicating the need for further studies with longer follow-up and larger numbers of patients [15].

The effectiveness of combined laser therapy was also evaluated by Jain and Jain [10] in a pilot study with 4 patients with HS and 5 with pilonidal sinus (PNS). Treatment began with a long-pulse Nd:YAG laser at 1064 nm to remove hair surrounding the lesions, followed by CO_2_ laser deroofing after 15 days. The time interval allowed for complete hair clearance. The average healing time was approximately 15 days. Additional laser hair removal sessions were performed every 2–3 months for a total of 4–5 sessions with no recurrence. Vivek and Archana noted that lasers used separately have been associated with relapses; therefore, they emphasized the effectiveness of combined therapy [10].

Lindén et al. [16] evaluated combined therapy CO_2_ and Ga-As laser in HS, as ablative fractional lasers enhance the penetration depth of non-ablative fractional lasers. Settings were adjusted to the Fitzpatrick skin type—I–III: AFL 16 W, 0.5 ms, and 8 mJ and NAFL 8 W, 6 ms, and 48 mJ; Skin type IV–VI: AFL 16 W, 0.25 ms, and 4 mJ and NAFL 8 W, 4 ms, and 32 mJ. All 8 patients showed clinical improvement, as evidenced by decreased active lesion count, diminished inflammatory response, lower pain, and fewer exacerbations. Lindén et al. highlighted that important benefits of mixed technology may be attributed to multiple mechanisms, such as decreasing the follicular inflammation, modification of the extracellular matrix, lowered sebum production, and follicular occlusion. These aspects may reduce biofilm formation [16].

In summary, the available data indicate that CO_2_ laser, both in the classical and scanner-assisted techniques, is an effective, safe, and well-tolerated surgical method for the treatment of HS Hurley stage II and III. It allows for selective tissue debulking while preserving healthy structures, ensures a low recurrence rate in the treated areas, and provides acceptable cosmetic results.

## 4. Neodymium Laser

Another promising therapy for HS is the Nd:YAG (neodymium-doped yttrium aluminum garnet; Nd: Y_3_Al_5_O_12_) laser. The therapeutic mechanism of Nd:YAG is thought to involve follicular ablation and the destruction of inflammatory lesions through photothermolysis. This treatment option is considered the most useful in Hurley stage I–II, because of its ability to reduce the number of hair follicles and sebaceous glands, and consequently to reduce the bacterial load [18].

In a study focusing on laser treatment for HS, Tierney et al. [19] emphasized the significance of this technique in managing the disease. A total of 22 patients with Hurley stage II to III HS underwent a series of treatments using a 1064 nm wavelength laser over a period of three months. Treatment outcomes were assessed after each laser session and again 1 month after therapy completion using the HS-LASI scale. After three months of treatment, the overall percentage reduction in HS severity was 65.3% across all anatomical sites, with reductions of 73.4% in the inguinal region, 62.0% in the axillary region, and 53.1% in the inframammary area. The improvement in HS severity from baseline to the third month was statistically significant at all treated sites (*p* < 0.02 for both the modified HS-LASI and original HS-LASI). In contrast, no significant change was observed at the control sites (*p* > 0.05 for both scales). The effectiveness of the long-pulse Nd:YAG laser, primarily used for hair epilation, in treating hidradenitis suppurativa supports the hypothesis of a primarily follicular pathogenesis of the disease [19].

Another clinically significant study addressing the topic is that by Mahmoud et al. [20]. Using the same 1064 nm Nd:YAG laser wavelength and pulse durations of 20 to 35 ms, the researchers treated a total of 22 patients with Hurley stage II HS. Progressive improvement in disease activity was observed, most notably during the 4-month treatment period, and this effect was maintained during the subsequent 2-month follow-up. Averaged across all anatomical sites, the mean percent improvement was 72.7% on the laser-treated side compared to 22.9% on the control side (*p* < 0.05). Histologic examination initially revealed an acute neutrophilic infiltrate, followed by granulomatous inflammation on biopsy specimens obtained four weeks later. An inflammatory infiltrate was observed surrounding remnants of hair shafts, indicating follicular destruction. Treatment response was assessed using the HS-LASI as described by Sartorius et al. Topical therapy with 10% benzoyl peroxide wash and 1% clindamycin lotion was applied to the control half of the body. At the same time, the laser-treated side received the same topical regimen in combination with the Nd:YAG laser. Patients underwent four monthly laser treatment sessions, with ultrasound gel applied to the skin before each procedure [20].

These findings clearly demonstrate that combining laser therapy with topical treatment yields significantly superior clinical outcomes compared to topical treatment alone.

A Castrillón Velásquez et al. [21] case involving a 40-year-old man with a 3-year history of chronic painful subcutaneous nodules, deep sinus tracts, and abscesses affecting the jawline and anterior neck region also led clinicians to consider treatment with the Nd:YAG laser. The patient had previously undergone multiple courses of topical and oral antibiotics, which resulted in only partial and temporary relief. Microbiological tests were performed to identify a potential infectious agent, but all results were negative. Isotretinoin at a dose of 10 mg daily was administered for 10 months without clinical improvement. Based on the clinical presentation, absence of an underlying infectious cause, and the lack of response to acne-targeted therapies, a diagnosis of hidradenitis suppurativa in an atypical location was established. The patient was also found to have insulin resistance [21].

Considering the severity of the condition (Sartorius score of 70 and Hurley stage II) and the lack of response to conventional treatments, biologic therapy with adalimumab (40 mg weekly) was initiated alongside isotretinoin. After three months, the patient showed significant improvement (Sartorius score reduced to 59), and isotretinoin was discontinued. Subsequently, monthly long-pulsed Nd:YAG laser treatment (1064 nm wavelength) was introduced as an adjuvant therapy to prevent hair follicle occlusion. After 1 year of combined adalimumab and laser therapy, the patient continued to improve, achieving a Sartorius score of 14 [21].

Similar results were reported by Fabbrocini et al. [18]. The study involved 20 patients aged 18–44 years with HS lesions in the axillae, groin, buttocks, brisket, and inframammary folds. All participants were non-pregnant and had no comorbidities. Half of the patients were classified as Hurley stage I, while the remaining half were at stage II; no patients with Hurley stage III were included. Each participant underwent four laser treatment sessions, one every two weeks. Before and after the treatment, patients were evaluated using the Hurley staging system, the Sartorius score, and the Physician Global Assessment (PGA). A standard energy fluence of 250 J/cm^2^ was used, with fiber diameters varying according to lesion type and anatomical location. The procedure was performed under local anesthesia with mepivacaine hydrochloride and epinephrine administered at the superficial subcutaneous plane. Starting on the day of the laser-surgical procedure, all patients received oral azithromycin 500 mg once daily for 3 days. By the end of the study, none of the patients experienced clinical worsening. Two showed no response to treatment, ten had a partial response, seven showed good improvement, and one patient had a complete response (>65% improvement). Most patients (18 out of 20) tolerated the procedure well without any symptoms. Reported adverse effects included postoperative pain, erythema, and mild swelling. One patient experienced fever and influenza-like symptoms, which resolved spontaneously [18].

In a prospective study, Xu et al. [22] evaluated the efficacy of a long-pulsed 1064 nm Nd:YAG laser in 20 patients with Hurley stage II HS. Treatment consisted of single laser pulses over affected areas, with additional stacking on inflamed lesions. Cooling gel and ice packs were used, without anesthesia. Laser parameters were adjusted according to Fitzpatrick skin type. Treatment response was assessed using the modified HS-LASI score, extended analogously to the versions used in the previously cited studies. Patients were randomized by anatomical site, with untreated areas serving as intraindividual controls. Serial skin biopsies were taken to monitor histopathological changes. After two months, a significant reduction in HS-LASI scores was observed: −31.6% overall (*p* < 0.001), −24.4% in the axillary region (*p* = 0.008), and −36.8% in the inguinal region (*p* = 0.001). While most patients improved both clinically and histologically, three showed persistent microscopic inflammation. The small sample size limited the interpretation of results in this subgroup [22]. The comparison of studies on HS treatment using the neodymium laser is presented in Table 2.

## 5. Alternative Laser- and Energy-Based Therapies in HS Management

### 5.1. Intense Pulsed Light Therapy (IPL) and Radiofrequency (RF)

Beyond the more extensively studied CO_2_ and Nd:YAG laser therapies, emerging light-based modalities are gaining attention in HS treatment studies. Among them, Intense Pulse Light (IPL) therapy has demonstrated clinical potential.

In managing hidradenitis suppurativa, IPL works by delivering high-energy light that penetrates the skin and targets the hair follicles. The generated heat causes controlled thermal damage, reducing follicular occlusion, which is a key factor in HS development. Additionally, IPL has an antibacterial effect—by activating bacterial porphyrins, it leads to the production of reactive oxygen species that kill bacteria, particularly Cutibacterium acnes. This dual action helps to ease inflammation and reduce the frequency and severity of HS flare-ups [23].

The prospective study by Highton et al. evaluated IPL in 17 HS patients diagnosed with HS, Hurley stage II or III. Patients were randomized to the treatment of one area with intense pulsed light therapy two times per week for 4 weeks. The contralateral affected sides not subjected to treatment served as controls. The results, analyzed based on clinical photographs and opinion of blinded plastic surgeons, showed a significant reduction in hidradenitis suppurativa severity on the treated side (*p* < 0.001), as well as little to no change on the control side. No complications such as blistering or pigmentation changes were observed. The patients were highly satisfied with the treatment [23].

Beyond the limited studies on the effectiveness of IPL in treating HS, attempts are being made to combine this type of therapy with others. One such option is the addition of radiofrequency (RF). Since non-ablative RF and IPL differ significantly in their mechanisms of action, combining them could be particularly effective in broadening the therapeutic spectrum for hidradenitis suppurativa.

RF generates heat directly in the deeper skin layers, independent of chromophores, meaning its efficacy is not dependent on skin color or pigmentation. It promotes collagen synthesis and tissue tightening while also reducing inflammation through thermal modulation of immune cell activity and increased blood circulation. This makes RF a well-suited addition following IPL’s antibacterial action, helping to support tissue healing and skin remodeling. This combination was explored by Wilden et al. in a group of 47 patients who were initially treated with either IPL monotherapy, RF monotherapy, or IPL + RF combination therapy (LAight^®^ therapy). After the initial 12 weeks of therapy, a crossover was implemented. During this phase, all patients, regardless of their initial group, received the combined IPL+RF therapy for an additional 12 weeks. Taking into consideration the photographic documentation, blinded dermatologist examination results, and Hurley score, the active lesion count in the IPL + RF group decreased more than in the IPL group (*p* = 0.044). Additionally, the reduction in DLQI reported by the patients was significantly greater in the IPL+RF and RF groups compared to the IPL group. The results were significantly better after 24 weeks of therapy compared to the first 12 weeks, demonstrating that prolonged treatment is superior to short-term therapy. Moreover, patients with earlier Hurley stages (I–II) showed a greater benefit from IPL + RF treatment compared to those with Hurley stage III. This suggests that this type of combination therapy may be more suitable for less advanced HS cases [24].

In another study by Schultheis et al., this approach was further explored, examining the efficacy of adding an IPL and RF mixed device to a 1% clindamycin solution. The study included 81 patients, all with HS stages I and II. One group received a combination of LAight^®^ therapy (IPL + RF) and topical clindamycin 1% solution, while the other group received only topical clindamycin 1% solution. After 16 weeks, the group treated with the combined therapy showed a 60% improvement in disease severity, compared to an average reduction of 17.8% in the group treated with clindamycin alone. Combination therapy also influenced the quality of life in patients, showing better results than monotherapy. The treatment was well-tolerated, with only mild and temporary side effects [25].

Lately, the LAight^®^ therapy has also been investigated in terms of effectiveness and safety in real-world clinical settings. Among 3437 patients diagnosed with HS I–III, during 26 weeks of care with LAight^®^, significant decreases in IHS4, pain-NRS, and DLQI were observed. The BMI at baseline had a significant adverse effect on therapy response for pain perception and general life quality. The therapy was generally well-tolerated, with most sessions completed without adverse effects, aside from occasional transient erythema (5.7%) and swelling (2.9%). The study concluded that LAight^®^ therapy is both effective and safe for treating HS across all disease stages [26].

Taking this into account, IPL appears to be the most effective monotherapy for more advanced hidradenitis suppurativa, while in milder cases, a combined approach with RF and/or clindamycin seems even more promising. However, although initial studies show encouraging results, further research is necessary to confirm the long-term efficacy and safety of the above-mentioned HS treatments and their relevance to application in different stages of the disease. The comparison of studies on HS treatment using IPL is presented in Table 3.

### 5.2. Diode Laser

Another treatment option for HS, increasingly studied for its potential benefits, is the diode laser. Unlike other therapies, the diode laser stands out because of its high precision and deep tissue penetration. The single, concentrated wavelength of light (800–980 nm) specifically targets melanin within hair follicles, leading to selective photothermolysis, which causes thermal destruction of the hair follicle. This mechanism can be particularly beneficial for reducing follicular occlusion and exerting antibacterial effects through its thermal impact—both of which are primary triggers of HS lesions—while also preventing recurrent follicular inflammation [27].

These effects were evaluated in the study by Özdemir et al. where 16 patients underwent two to four sessions of 808 nm diode laser therapy. They experienced a noticeable decrease in disease severity, as indicated by improvements in clinical scores. A significant reduction in symptoms and improvement in quality of life were observed. The treatment was well tolerated, with only mild pain and no severe adverse effects [27].

Another study by Brown et al. focused on a minimally invasive technique that uses laser energy to target sinus tracts and promote their closure, with energy settings adjusted based on lesion depth. The energy settings were adjusted according to lesion depth—8 Watts for deeper lesions and 5 Watts for superficial ones. The procedure was performed under local anesthesia for less extensive lesions and general anesthesia for more severe cases, with patients experiencing minimal postoperative pain and often returning to work quickly. This technique has shown promise, particularly in early-stage HS (Hurley stage I), but may require multiple sessions or adjunctive therapies for more advanced stages [28].

It is emphasized that combining laser treatment with other therapies, such as antibiotics and biologics, may be necessary for more extensive or refractory cases. Overall, the approach appears effective in managing HS while minimizing tissue damage. The summary of studies on HS treatment using diode laser is presented in Table 4. 

### 5.3. Alexandrite Laser

The Alexandrite laser (755 nm) has been shown to be promising for the treatment of hidradenitis suppurativa. Melanin, as the target molecule, absorbs the emitted light, leading to thermal destruction of the follicle and consequently eliminating follicular occlusion—the primary mechanism contributing to HS flare-ups [29].

For instance, the study by Sidhom et al., involving patients with bilateral, symmetric HS, reported a 75% clinical response rate in treated areas compared with 33.33% in untreated control sites [27]. Another study with women in Hurley stage I or II HS found that 70% of patients achieved a clinical response following several laser sessions, alongside improvements in pain levels and quality of life [30].

The main advantage of the Alexandrite laser lies in its precision, as it specifically targets hair follicles while sparing the surrounding healthy tissue. It offers a minimally invasive approach, making it a safer alternative to more invasive surgical treatments and reducing the risk of scarring. Additionally, it can be combined with other therapies, such as antibiotics or biologics, to enhance overall efficacy.

Although this laser is characterized by its precision and ability to selectively target melanin in hair follicles, thereby avoiding surrounding healthy tissue, it is most effective in individuals with lighter skin types (Fitzpatrick I–III). Moreover, multiple treatment sessions may be necessary to achieve the desired outcomes, particularly in more severe or recurrent cases of HS. In conclusion, the Alexandrite laser shows promise as an adjunctive therapy for managing HS, especially in cases involving follicular occlusion, but further research is needed to establish its long-term efficacy. The summary of studies on HS treatment using alexandrite laser is presented in Table 5.

## 6. Conclusions

**Figure 2:**
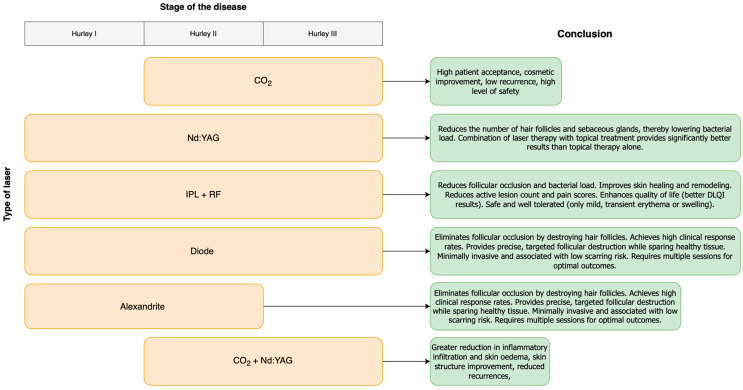
Summary of the use of laser therapy in HS.

Laser therapy represents a valuable adjunct and, in some cases, a primary treatment modality for patients with HS, particularly those who are refractory to medical management or experience recurrent disease (Figure 2). Among the laser-based approaches analyzed, CO_2_ laser therapy stands out as the most extensively studied and clinically effective option, particularly for patients with Hurley stage II and III disease. It allows for precise excision or vaporization of affected tissue, facilitates healing by secondary intention, and demonstrates a relatively low recurrence rate in treated areas, with high patient satisfaction and acceptable cosmetic outcomes.

Long-pulsed Nd:YAG lasers also show significant promise, especially in the early stages of HS (Hurley I–II). They achieve improvement through follicular ablation and photothermolysis, reducing inflammation and disease activity. Multiple studies demonstrated statistically significant reductions in HS severity scores when Nd:YAG laser treatment was used alone or in combination with topical therapy, especially clindamycin.

Emerging modalities such as IPL and RF, particularly in combination (e.g., LAight^®^ therapy), also show encouraging results in reducing lesion count and disease severity and in improving quality of life—especially in early-stage HS. These therapies are well-tolerated and may offer a non-invasive alternative for patients unsuitable for surgical intervention.

Additional energy-based treatments, such as the diode laser and alexandrite laser, offer targeted follicular destruction with favorable safety profiles and promising early outcomes. These therapies may be especially beneficial for patients with limited or localized disease and those who are seeking minimally invasive options.

Importantly, combination strategies—whether involving multiple laser types or pairing laser therapy with pharmacologic treatments—appear to enhance therapeutic outcomes, reduce recurrence risk, and broaden the scope of individualized HS management. Factors such as disease stage, lesion location, patient comorbidities (e.g., obesity), and skin type influence therapeutic efficacy and should guide treatment selection.

Overall, laser- and energy-based therapies represent a safe and effective component of the multidisciplinary approach to HS treatment. In addition to its clinical benefits, laser therapy appears to improve patient-reported outcomes, including pain reduction and overall quality of life. However, further randomized controlled trials with larger patient populations, more extended follow-up periods and comparator studies of different lasers are needed to standardize treatment protocols, clarify long-term outcomes, and establish consensus guidelines for integrating these modalities into routine clinical practice.

## Figures and Tables

**Table 1:** Comparison of studies on HS treatment using CO_2_ laser.

Author (Year)	Population/Disease Duration/Hurley Stage	Laser Settings and Sessions (Parameters and Technique; Sessions)	Healing Time and Time of Follow-Up	Outcome	Adverse Events
Lapins et al. (1994) [5]	21 F; 3 M/1–28 years/ Hurley II–III	30 W, paintbrush technique; 1 session/area	3–5 weeks; 15–47 months	33% RF 8% recurrence	Secondary infections in 8%
Finley and Ratz (1996) [6]	7 F/1–10 years/ND	40 W, continuous wave; 12 procedures (5 bilateral, 2 unilateral)	4–8 weeks; 10–27 months	14% recurrence	Temporary paresthesias in 14%; axillary stricture in 14%; inguinal candidiasis in 14%
Lapins et al. (2002) [7]	31 F; 3 M/1–35 years/Hurley II	20–30 W, continuous wave; 67 operation sites	3–5 weeks; 7–87 months	24% RF; 12% recurrence	Temporary axillary paresthesia in 3%
Madan et al. (2008) [8]	8 F; 1 M/4–17 years/severe, multifocal HS	15–25 W, ultrapulse mode; 27 sites in 19 sessions	1–4 weeks; 12 months	67% RF	Axillary contracture scar in 22% (not limiting); dehiscence of the wound in 11%
Hazen and Hazen (2010) [9]	42 F; 19 M/1–25 years/advanced, recurrent HS	8–30 W, marsupialization; 154 sessions	Average of 8.8 weeks; 1–19 years	1–17 years RF in treated areas; 3% recurrence	Granulation tissue in 28%; cellulitis in 5%; Sweet’s syndrome in 2%; dehiscence of the wound in 2%
Jain and Jain (2012) [10]	4 F/ND/ND	Nd:YAG laser 1064 nm (30 J, 30 ms, 10 mm spot size) CO_2_ laser deroofing (30 W)	15 days; 3 years	0% recurrence	ND
Krakowski et al. (2014) [11]	1 adolescent F/1 year/post-HS scarring	Fractionated CO_2_ (15 mJ, 15% density); 2 sessions	2 days; 6 months	Cosmetic improvement; 0% recurrence	ND
Mikkelsen et al. (2015) [12]	48 F; 10 M/4–39 years/ND	20–35 W and 4 mm spot size	ND; 1–47 months	29% recurrence	ND
Crocco et al. (2016) [13]	2 F; 1 M/2–10 years/Hurley III	30 W, dynamic mode; 1–3 sessions/area	(1) 9 weeks: ND (2) ND; 6 months (3) ND; ND	100% RF; 0% recurrence	ND
Nicholson et al. (2016) [14]	1 F/ND/Hurley III	50 J, 5% density, 120 μm spot size; 4 sessions	ND; 16 months	Cosmetic improvement; 0% recurrence	ND
Abdel Azim et al. (2018) [15]	11 F; 9 M/2–10 years/Hurley II–III	Fractional CO_2_ laser (15 W, 10,600 nm) + Nd:YAG (1064 nm); 80 sessions (4 sessions/patient with 2 weeks intervals)	ND; at 2 weeks and 3 months	(1) Combined treatment: 80% RF (2 weeks), 55% RF (3 months) (2) Nd:YAG only: 35% RF (2 weeks), 20% RF (3 months)	Transient erythema that disappeared within 48 h
Lindén et al. (2022) [16]	5 F; 3 M/1–42 years/Hurley II–III	Skin type-I–III: CO_2_ 16 W, 0.5 ms, 8 mJ and Ga-As 8 W, 6 ms, 48 mJ; Skin type IV–VI: CO_2_ 16 W, 0.25 ms, 4 mJ and Ga-As 8 W, 4 ms, 32 mJ	ND; ND	ND	Minor and transient burning and redness in 100%
Sechi et al. (2025) [17]	3 F; 7 M/ND/Hurley II	4–10 W; continuous wave mode	3–7 weeks; 6 months	10% recurrence	Bleeding in 10%

F—female; M—male; RF—recurrence free; ND—not defined.

**Table 2:** Comparison of studies on HS treatment using the neodymium laser.

Author (Year)	Population/Disease Duration/Hurley Stage	Laser Settings and Sessions (Parameters and Technique; Sessions)	Healing Time and Time of Follow-Up	Outcome	Adverse Events
Tierney et al. (2009) [19]	19 F; 3 M/ND/Hurley II–III	Long-pulsed Nd:YAG; 1064 nm/3 monthly sessions	ND, 4-week follow-up	65.3% overall HS-LASI reduction; statistically significant only at treated sites (*p* < 0.02)	ND
Mahmoud et al. (2010) [20]	19 F; 3 M/ND/Hurley II	Long-pulsed Nd:YAG; 1064 nm; 20–35 ms/ 4 monthly sessions;	ND, 8-week follow-up	72.7% improvement (laser) vs. 22.9% (control)	ND
Castrillón Velásquez et al. (2017) [21]	1 M/3-year duration/Hurley II	Long-pulsed Nd:YAG; 1064 nm/12 monthly sessions	ND, 52-week follow-up	Sartorius score improved from 70 to 14;	ND
Fabbrocini et al. (2018) [18]	14 F; 6 M/ND/Hurley I–II	Long-pulsed Nd:YAG; 1064 nm; 250 J/cm^2^/4 sessions every 2 weeks	ND, follow-up after last session included	1 complete, 7 good, 10 partial, 2 no response; no worsening in any case	Mild pain, erythema, swelling; 1 case of fever (self-limited)
Xu et al. (2011) [22]	17 F; 3 M/ND/Hurley II	Long-pulsed Nd:YAG; 1064 nm; stacking pulses; adjusted to skin type	ND, 8.5-week follow-up	HS-LASI score reduced by −31.6% overall	ND

F—female; M—male; ND—not defined.

**Table 3:** Comparison of studies on HS treatment using IPL.

Author (Year)	Population/Disease Duration/Hurley Stage	Laser Settings and Sessions (Parameters and Technique; Sessions)	Healing Time and Time of Follow-Up	Outcome	Adverse Events
Highton et al. (2011) [23]	15 F, 3 M/ND/Hurley II–III	Intense Pulse Light–Harmony Laser 420 nm; fluence, 7 to 10 J/cm^2^; pulse width, 30 to 50 msec; 8 sessions (2 per week for 4 weeks)	ND; ND	Significant improvement on the treated side in the mean examination score maintained at 12 months, high patients’ satisfaction	ND
Wilden et al. (2021) [24]	47 MF/at least 6-month duration/at least 3 abscesses or nodules	IPL + RF combination or IPL/RF monotherapy for 12 weeks followed by 12 weeks IPL + RF combination for all	Total treatment time: 24 weeks (2 × 12-week phases); follow-up not extended beyond treatment period	Change in active lesions −3.6 (*p* = 0.001); DLQI −5.2 (*p* = 0.003); After 12 weeks active lesions of the IPL + RF group decreased more than in the IPL group	wound healing delay (*n* = 3), transient mild to moderate pain (*n* = 2) lasting less than 12 h, a transiently elevated skin sensitivity (*n* = 2), a transient mild fever (*n* = 1), and a regional numbness (*n* = 1)
Schultheis et al. (2022) [25]	88 MF/ND/Hurley I–II	LAight^®^ therapy (IPL + RF); 8 sessions over 16 weeks (bi-weekly)	Total treatment time: 32 weeks First follow-up at week 16	ΔIHS4: −7.2 (−60%) vs. −1.8 (−17.8%), *p* < 0.001; combined treatment LAight^®^ + clindamycin superior to monotherapy—significantly higher decrease in disease severity and improvement in quality of life	Erythema (*n* = 26), edema/swelling (*n* = 18), blisters/crustification (*n* = 11), other AE (*n* = 2)
Strobel et al. (2024) [26]	2 282 F, 1 155 M/ND/Hurley I–III	LAight^®^ therapy (IPL + RF); session frequency not specified	26-week treatment period; 6 months follow-up	Significant reductions in IHS4, pain-NRS, and DLQI across all Hurley stages; pain response rates at week 26: 80.0% (Hurley I), 70.6% (Hurley II), 42.8% (Hurley III); DLQI response rates: 66.4% (Hurley I), 61.3% (Hurley II), 52.1% (Hurley III)	Mild, transient erythema (5.7%) and edema (2.9%)

F—female; M—male; RF—radiofrequency; ND—not defined; IPL—intense pulse light; AE—adverse events; DLQI—Dermatology Life Quality Index; IHS—International Hidradenitis Suppurativa Score System; NRS—numerical rating scale.

**Table 4:** Comparison of studies on HS treatment using diode laser.

Author (Year)	Population/Disease Duration/Hurley Stage	Laser Settings and Sessions (Parameters and Technique; Sessions)	Healing Time and Time of Follow-Up	Outcome	Adverse Events
Brown et al. (2025) [28]	32 MF/mean duration 8.7 yrs/Hurley I–III	1470 nm diode laser; 5 W for shallow lesions, 8 W for deep lesions; session frequency not specified	ND; ND	Minimally invasive technique focusing on tissue preservation; emphasized importance of multidisciplinary approach and close follow-up	ND
Özdemir and Tamer (2024) [27]	16 patients (13 men, 3 women), aged 23–61/ND/ND	808 nm diode laser; 2–4 sessions; no systemic treatment 3 months prior or during therapy	ND; 6-month follow-up	Significant reduction in HS severity (HS-PGA 3.0 → 2.0, *p* = 0.012); 8/10 patients responded; improved DLQI (4.5 → 1.0, *p* = 0.002)	Mild pain; no severe adverse effects

F—female; M—male; ND—not defined; DLQI—Dermatology Life Quality Index; HS-PGA—Hidradenitis Suppurativa Physician’s Global Assessment.

**Table 5:** Comparison of studies on HS treatment using alexandrite laser.

Author (Year)	Population/Disease Duration/Hurley Stage	Laser Settings and Sessions (Parameters and Technique; Sessions)	Healing Time and Time of Follow-Up	Outcome	Adverse Events
Sidhom et al. (2024) [29]	15 adult patients (age 18–60) with bilateral symmetric HS/ND/ND	755 nm alexandrite laser, 4 monthly sessions on one side;	Total treatment time: 24 weeks (4 monthly sessions); Follow-up: 8 weeks post-treatment	HiSCR response: 75% in treated sites vs. 33.3% in control (*p* = 0.0046); lesion improvement: axilla 72.7%, inguinal 70%, inframammary 100%	ND
Molinelli et al. (2022) [30]	40 F/ND/Hurley stages I–II	755 nm Alexandrite laser; 5 sessions at 6-week intervals	Total treatment time: 30 weeks; Follow-up after 15 and 30 weeks	HiSCR achieved in 70% of treated patients vs. 20% in control group. Reduction in acute flares and significantly longer disease-free survival in the treated group	ND

F—female; M—male; ND—not defined; HiSCR—Clinical Response.

## Data Availability

Data sharing is not applicable to this article as no datasets were generated or analyzed.

## Supplementary Materials

The following supporting information can be downloaded at: https://www.mdpi.com/article/10.3390/jcm14217683/s1, Table S1: PRISMA 2020 checklist; Table S2: PRISMA 2020 for Abstracts checklist. Reference [31] is cited in the supplementary material.
